# Equality of healthcare resource allocation between impoverished counties and non-impoverished counties in Northwest China: a longitudinal study

**DOI:** 10.1186/s12913-024-11312-5

**Published:** 2024-07-22

**Authors:** Liang Zhu, Wei Gao, Siyu Zhang, Fei Yu, Jiaxue Li, Junqiang Feng, Rui Wang

**Affiliations:** 1 Department of Health Service Management and Medical Education, School of Public Health, Air Force Medical University, No. 169, Changle West Road, Xincheng District, Xi’an, Shaanxi Province 710032 China; 2 The Ministry of Education Key Lab of Hazard Assessment and Control in Special Operational Environment, Xi’an, Shaanxi 710032 China; 3 Teaching Archives Room, Air Force Medical University, Xi’an, Shaanxi 710032 China; 4 Teaching and Evaluating Center, Air Force Medical University, Xi’an, Shaanxi 710032 China; 5 Xi’an International Medical Center Hospital, Xi’an, Shaanxi Province 710000 China; 6 Library of Teaching and Research Support Center, Air Force Medical University, Xi’an, Shaanxi 710032 China

**Keywords:** Health resource allocation, Equality, Gini coefficient, Theil Index, Health Resource Agglomeration Degree

## Abstract

**Background:**

The Health and Medical Assistance Program for Poverty Alleviation is part of China’s targeted poverty elimination strategy, which aims to protect poor people’s right to health and prevent them from becoming trapped in or returning to poverty because of illness. Many tasks have been defined in this program, including raising the medical insurance level, providing a triage system, improving medical and health services, and enhancing people’s health. One pivotal aspect of this initiative involves equitable health resource allocation, a key measure aimed at bolstering medical and health services. This study aimed to analyze and compare health resource allocations in different counties in Northwest China after the implementation of the program.

**Methods:**

The Gini coefficient quantifies the level of distributional equality, the Theil index assesses the sources of inequality, and the Health Resource Agglomeration Degree gauges the accessibility of health resources.

**Results:**

1) The health resource allocation distributed based on population(Gini Coefficient < 0.45) was more equitable than that distributed based on area(Gini Coefficient > 0.35) among counties in Northwest China. 2) The contribution rate within non-impoverished counties is higher than that of impoverished counties, which means the inequality within non-impoverished counties. 3) The allocation of beds in medical institutions by area in non-impoverished counties was better than that in impoverished counties, and accessibility to health services for residents in non-impoverished counties was better than that in impoverished counties.

**Conclusion:**

The analysis of health resource allocation among the five provinces in Northwest China revealed significant differences in equality among the five provinces in Northwest China, and the differences were mainly derived from the non-impoverished counties. Although the equality is gradually improving, the number of health resources in impoverished counties remain lower than that in non-impoverished counties.Subsequently, it is essential to ensure equitable distribution of healthcare resources while also taking into account their utilization and quality.

**Supplementary Information:**

The online version contains supplementary material available at 10.1186/s12913-024-11312-5.

## Background

The World Health Organization (WHO) health policy aims to ensure equitable health service provision, which is a key aspect in achieving population health fairness [1]. Evidence suggests that equitable health-resource allocation is crucial in providing fair health service [2], particularly for vulnerable populations [3]. An ideal allocation of healthcare resources gives people access to the same healthcare service without geographical and/or economical difference [4]. Despite governments’ dedication to health resource allocation, disparities in access to healthcare persist as a prevalent characteristic in both wealthy and poor countries. In general, poor and minority populations with the lowest health statuses have limited access to healthcare, whereas the rich receive more and better quality services [5]. A lack of access to quality healthcare exacerbates poverty and can result in mortality [6].

A growing body of research on the equality of healthcare resource allocation has examined both demographic and geographic equality, with the common consensus being that demographic equality is better than geographic equality [7]. The evaluation methods for health resource equality in the literature usually include the Gini Coefficient, Theil Index, Health Resources Agglomeration Degree, Index of Dissimilarity, and Moran’s I [8]. The existing literature on healthcare resource allocation in various cities emphasizes the influence of economic development levels, underscoring the significant variation in healthcare systems across different regions [9]. For example, medical staffing levels are lower in poor counties [10]. Furthermore, rural areas face healthcare allocations inequality [11]. Overall, healthcare allocation is unbalanced in several aspects; however, such studies remain narrowly focused, dealing only with one type of resource or resources at the provincial and municipal levels.

Chinese governments launched the healthcare Program for Poverty Alleviation in 2016 to prevent the poor from sinking back into poverty because of illness. The aim of program is to enable the public to access reduced medical consultations or even forgo them altogether, while still receiving high-quality healthcare and being able to afford medical treatment [12].To guarantee access to essential medical services for the poor, governments have taken comprehensive measures, including elevating the medical insurance level, improving medical and health services, and enhancing the population health levels in poor areas. Key counties are included in the national plan for poverty alleviation through development to meet the “three one” target—one public county-level hospital for each county, one standardized township health centers for each town, and one clinic for each administrative village [13]. As of November 2020, all 832 impoverished counties in China, including 196 in the northwest region, have emerged from poverty, indicating that China had eliminated absolute poverty [14]. Aligning efforts to consolidate the results of the battle against poverty with rural revitalization was proposed in the *No.1 Central Document* and *Government Work Report* of 2021 (https://english.www.gov.cn/2021special/govtworkreport2021). One of the measures implemented by the government is optimizing the allocation of healthcare resources in impoverished areas to promote residents’ health.

Although the main measure of health poverty alleviation is the policy of medical security, health resources are important factors in the provision and utilization of health services. Northwest China, with its high poverty concentration, uneven allocation of health resources, policy focus and other characteristics, has become a typical region to study the theme of health resources and poverty. Therefore, this study measures the counties’ health resource allocation by population and geography in Northwest China from 2015 to 2019, and compares the equality and temporal changes between impoverished and non-impoverished counties, to optimize health resource allocation in Northwest China.

## Methods

### Sample selection

Before selecting samples, we first needed to clarify two concepts: Northwest China, characterized by a relatively small population and an underdeveloped economy, comprises Shaanxi Province, Gansu Province, Qinghai Province, Ningxia Hui Autonomous Region, and Xinjiang Uygur Autonomous Region. The impoverished counties in this study refer to the national-level poor counties identified in 2015, which were defined based on the average annual net income of the local people (832), all of which have been lifted out of poverty in 2020.

The sampling process was divided into three steps. Firstly, we identified the geographical indications for the five provinces. We chose the geographical features to divide the five provinces because poverty is highly related with geographic location [15], and each province has a regional standard that is based on specific terrain. For example, we could divide Shaanxi Province into northern Shaanxi, the Guanzhong area, and southern Shaanxi according to the Qinling Mountains and the Daba Mountains. Secondly, we proceeded with a random stratified sampling approach to select impoverished counties from each of the five provinces in Northwest China, ensuring a proportion of 20% based on the geographical divisions. Thus we chose 12 impoverished counties in Shaanxi Province, 11 impoverished counties in Gansu Province, 2 impoverished counties in the Ningxia Hui Autonomous Region, and 5 impoverished counties in the Xinjiang Uygur Autonomous Region. Thirdly, we also selected the same number of non-impoverished counties with similar GDP as far as possible from the five provinces. Notably, Qinghai Province had four non-impoverished counties only, necessitating the selection of four additional impoverished counties to maintain balance in the sampling process.

Thus, we selected 68 counties in total as survey sample, including 24 counties from Shaanxi Province, 22 from Gansu Province, 8 from Qinghai Province, 4 from Ningxia Hui Autonomous Region, and 10 from Xinjiang Uygur Autonomous Region. A list of counties selected in each group in each province is presented in the appendix.

### Data collection and indicators

Based on the requirements of objectivity, representation, availability, stability, and consistency in a previous study [16], the numbers of medical personnel and beds in medical institutions were included. Medical personnel can represent an investment in human resources, including medical practitioners, assistant medical practitioners, registered nurses, pharmacists, laboratory technologists, imaging technologists, health supervisors, and medical trainees. Beds in medical institutions can represent investments in material resources. Additionally, to ensure comparability, the values of these variables were calculated per 1000 population, utilizing data on the number of permanent residents and the area of the prefectures.

This study used data from the *Statistical Report of National Economic and Social Development* of each county, *China Statistical Yearbook(County-level),* and the *Chinese Statistical Yearbook*. *Statistical Report of National Economic and Social Development*, issued by County Bureau of Statistics, gathered data on a wide range of topics, including economic development, agriculture, industry, healthcare services, demographic. We extracted the number of permanent population at the end of a year, county and city areas, number of medical personnel and number of beds in medical institutions. *China Statistical Yearbook(County-level)* and *Chinese Statistical Yearbook*, issued by National Bureau of Statistics of China, contains information on the basic situation, comprehensive economy, agriculture, and other aspects. We supplemented the missing data from these two kinds of books.

The global outbreak of COVID-19 in 2020 has an impact on the utilization of health resources. Therefore, we used time-series data (2015–2019) to analyze the equality gap between the two kinds of counties.

### Gini coefficient

The Gini Coefficient is a widely used analysis indicator in measuring the social income gap of residents in a country (or region). It reflects the overall inequality, and ranges from 0 to 1 with 0.4 as a “warning line” for the gap. The significance of the Gini coefficient warning line is to remind the government and society to pay attention to and take measures to reduce income inequality, promote social equality and stability. Specifically, Gini Coefficient > 0.5 indicates “a huge gap”, 0.4 < Gini Coefficient < 0.5 denotes “a large gap”, 0.2 < Gini Coefficient < 0.3 means “relatively average”, and Gini Coefficient < 0.2 represents “absolute even” [17].

### Theil Index

The Theil Index is applied to measure equality and thus explain the sources of inequality, which could be divided into “within group” and “between group” [16]. It reveals whether the difference in health resource allocation by population emerges from the impoverished counties, non-impoverished counties, or from within both.The smaller the value, the greater the equality.

The calculation formula for the evaluation of health resources within impoverished or non-impoverished counties is

$${I}_{A}=\sum {g}_{Ai}log(\frac{{W}_{A}}{{W}_{i}})$$

In the above formula, ${I}_{A}$ represents inequality index of impoverished or non-impoverished counties, ${g}_{Ai}$ represents the ratio of one impoverished or non-impoverished county population to the population of total sampled impoverished or non-impoverished counties, ${W}_{A}$ represents the ratio of resources to population in total sampled impoverished or non-impoverished counties, ${W}_{i}$ represents the ratio of resources to population in one impoverished or non-impoverished counties [18].

The calculation formula for the evaluation of health resources between impoverished and non-impoverished counties is

$$I_{\mathit L}\mathit=G_{\mathit A}log\mathit(\frac{{\mathit G}_{\mathit A}}{{\mathit T}_{\mathit A}}\mathit)\mathit+G_{\mathit B}log\mathit(\frac{{\mathit G}_{\mathit B}}{{\mathit T}_{\mathit B}}\mathit)\mathit+\mathit\cdots\mathit+G_{\mathit N}log\mathit{\left(\frac{G_N}{T_N}\right)}$$

In the above formula, ${I}_{L}$ represents inequality index of impoverished and non-impoverished counties, ${G}_{A}$,${G}_{B}$,…,${G}_{N}$ represents the ratio of population of total sampled impoverished or non-impoverished counties to population of one province(region), ${T}_{A}$,${T}_{B}$,…,${T}_{N}$ represents the ratio of health resources of total sampled impoverished or non-impoverished counties to health resources of one province(region).

The formula for the Theil index is

$$I\mathit=I_{\mathit L}\mathit+G_{\mathit A}I_{\mathit A}\mathit+G_{\mathit B}I_{\mathit B}\mathit+\mathit\cdots\mathit+G_{\mathit N}I_{\mathit N}$$

In the above formula, $\text{I}$ represents the Theil index.

The contribution rate of differences within impoverished or non-impoverished counties is

$$I_{within\;group}=G_AI_A/I$$

The contribution rate of differences between impoverished and non-impoverished counties is

$$I_{between\;groups}=I_L/I$$

### Health Resource Agglomeration Degree

The Health Resource Agglomeration Degree (HRAD) indicates the concentration degree of health resource allocation in different regions. It refers to the proportion of health resources concentrated in a certain region’s land area, occupying 1% of the area of the upper and larger regions to which the region belongs to [19]. It reflects the degree of health resource agglomeration in a region, relative to a larger region [20], and its formula is as follows:

$${HRAD}_{i}=\frac{({HR}_{i}/{HR}_{n})\times 100\%}{({A}_{i}/{A}_{n})\times 100\%}=\frac{{HR}_{i}/{A}_{i}}{{HR}_{n}/{A}_{n}}$$

In the above formula, ${HRAD}_{i}$ represents the agglomeration degree of health resources in each county. ${HR}_{i}$ represents the health resource quantity of each county. ${A}_{i}$ represents the land area of each county. ${HR}_{n}$ represents the health resource quantity of the city (or autonomous prefecture) to which each county belongs, and ${A}_{n}$ represents the total land area of the city (or autonomous prefecture) to which each county belongs.

When ${HRAD}_{i}>1$, the county is relatively rich in health resources by geographical allocation; when ${HRAD}_{i}=1$, the health resources of the county are absolutely balanced by geographical allocation; when ${HRAD}_{i}<1$, the county is relatively short of health resources by geographical allocation [21].

Population Agglomeration Degree (PAD) evaluates the population density in a region. It refers to the proportion of the population gathered in a certain region’s land area, occupying 1% of the area of the upper and larger region to which it belongs, and is calculated as follows:

$${PAD}_{i}=\frac{({P}_{i}/{P}_{n})\times 100\%}{({A}_{i}/{A}_{n})\times 100\%}=\frac{{P}_{i}/{A}_{i}}{{P}_{n}/{A}_{n}}$$

In the above formula, ${PAD}_{i}$ represents the population agglomeration degree of each county, ${P}_{i}$ represents the population quantity of each county, and ${P}_{n}$ is the population of the city (or autonomous prefecture) each county belongs to [22].

We combined HRAD and PAD to evaluate equality in health resource allocation. When ${HRAD}_{i}-{PAD}_{i}$=0, the allocation of health resources in the county basically meets the needs of the local population, and the residents have good access to health services; when ${HRAD}_{i}-{PAD}_{i}>$ 0, the county has an excess of health resource allocation relative to the local population size, and the residents have better access to health services; when ${HRAD}_{i}-{PAD}_{i}<$ 0, the allocation of health resources in the county cannot meet the needs of the local population, and the residents’ access to health services is poor [23].

## Results

### Health resource allocation among counties in Northwest China

**Fig. 1:**
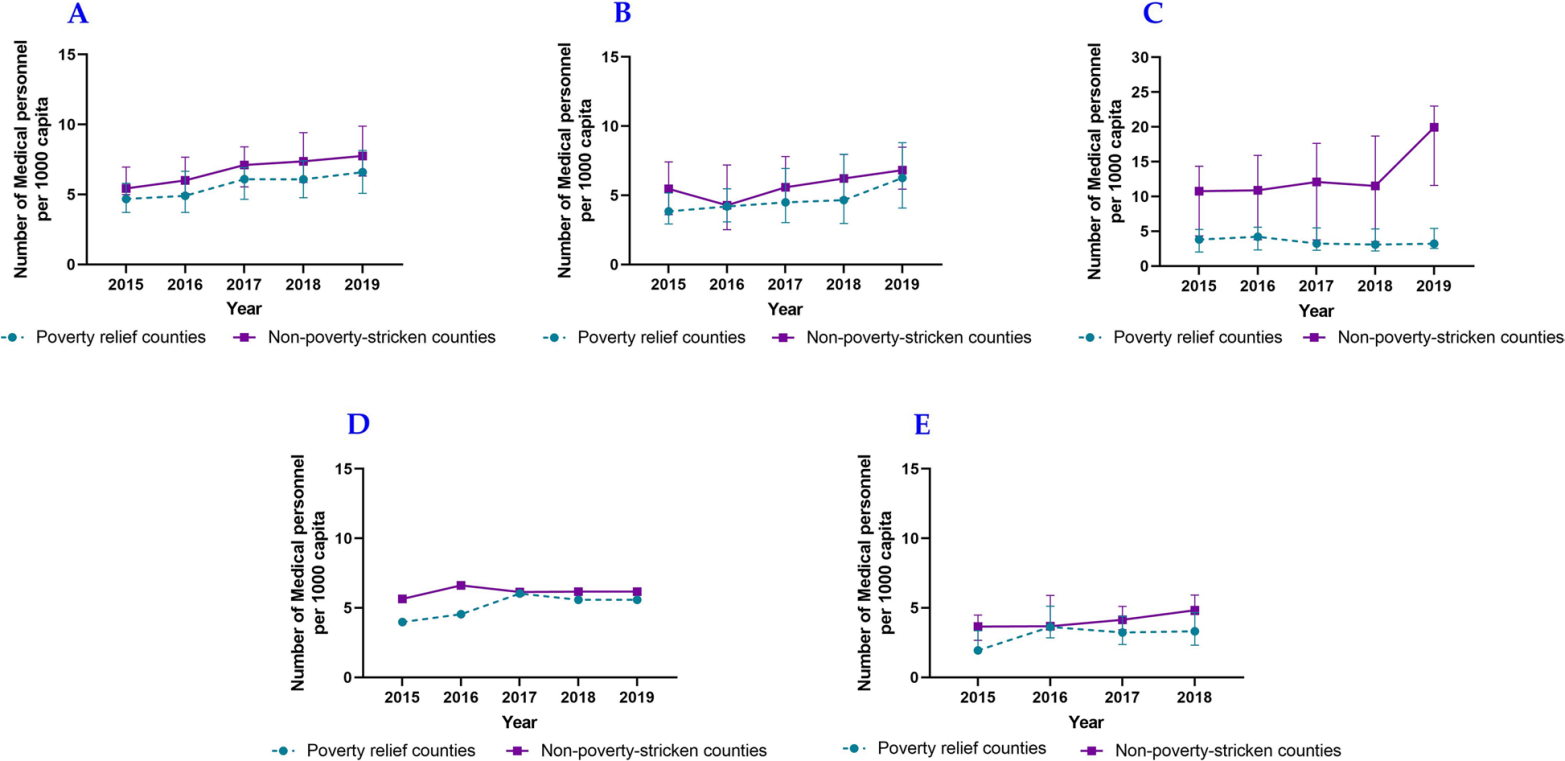
Medical and health personnel per 1000 capita of counties in Northwest China, including Shaanxi Province (**A**), Gansu Province (**B**), Qinghai Province (**C**), Ningxia Hui Autonomous Region (**D**), Xinjiang Uygur Autonomous Region (**E**)

**Fig. 2:**
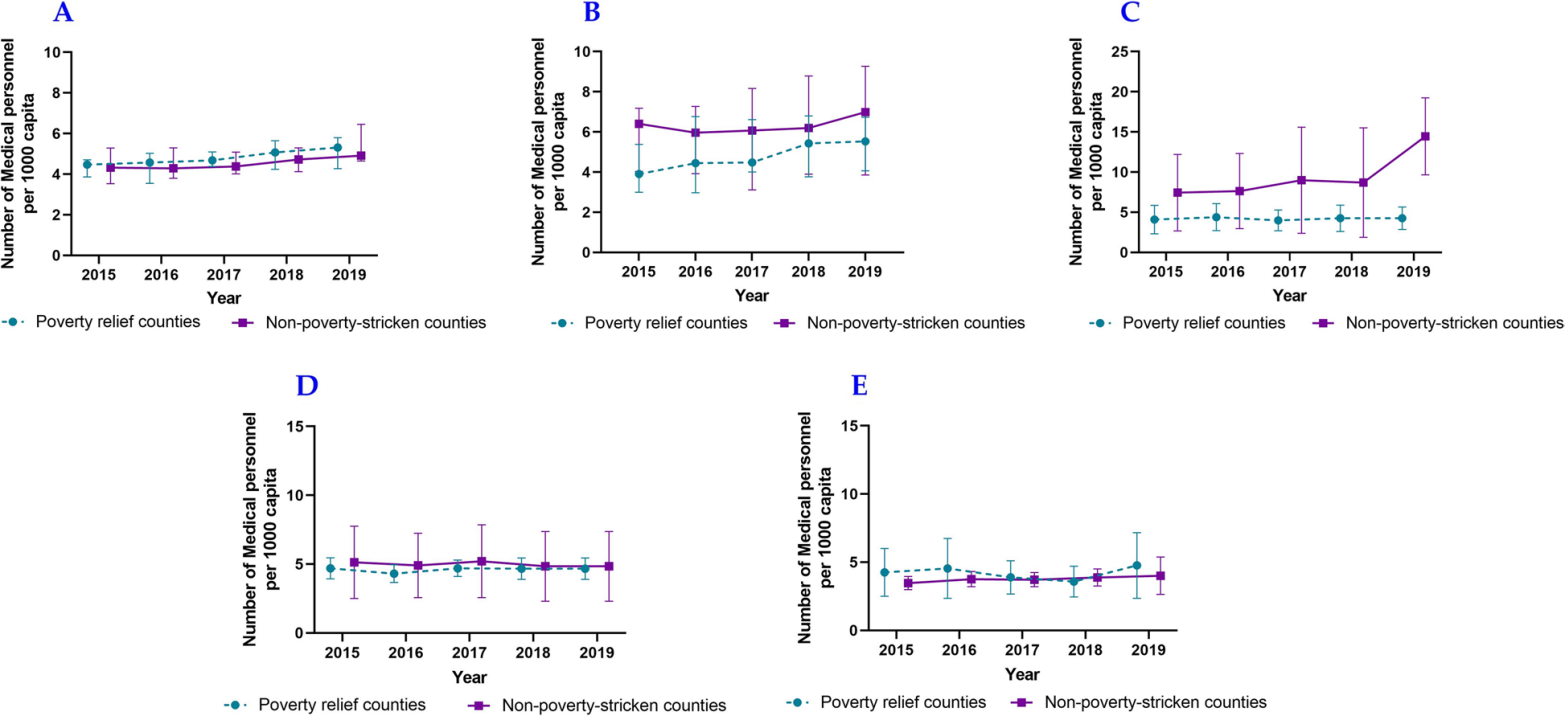
Beds in medical institutions per 1000 capita of counties in Northwest China, including Shaanxi Province (**A**), Gansu Province (**B**), Qinghai Province (**C**), Ningxia Hui Autonomous Region (**D**), Xinjiang Uygur Autonomous Region (**E**)

In this study, we used the median of the sample to represent health resource allocation. According to the 2015 ~ 2019 data, the number of medical personnel and beds in medical institutions per 1000 capita increased steadily, with the number of personnel growth being faster than that of beds. By comparison, the health resource allocation of impoverished counties was worse than that of non-impoverished counties. Regarding impoverished counties, Shaanxi Province had the largest number of medical personnel per 1000 capita, and Gansu Province had the highest number of beds in medical institutions per 1000 capita among five provinces. Regarding non-impoverished counties, Qinghai Province had the largest health resource allocation (Figs. 1 and 2).

### Equality in health resource allocation based on Gini coefficient

By calculating the Gini Coefficient of health resource allocation among counties in each province from 2015 to 2019, the values were below 0.50 by population and below 0.90 by area, suggesting the overall high inequality of health resource allocation by area.

Qinghai Province had the largest values by population, which reached “a large gap,” while the rest reached “relatively average” and “absolute even.” The values by area of the Xinjiang Uygur Autonomous Region were below 0.40, and the rest were above 0.50, which indicated considerable inequality in health resource allocation distributed by area (Table 1).

**Table 1:** The Gini coefficient of health resource allocation in five provinces from 2015 to 2019

Province	Year	By population		By area	
		Number of medical personnel	Number of beds	Number of medical personnel	Number of beds
Shaanxi	2015	0.19	0.20	0.61	0.61
	2016	0.25	0.22	0.62	0.59
	2017	0.20	0.20	0.60	0.59
	2018	0.20	0.20	0.61	0.59
	2019	0.18	0.17	0.60	0.58
Gansu	2015	0.25	0.23	0.61	0.58
	2016	0.27	0.21	0.59	0.58
	2017	0.27	0.23	0.61	0.57
	2018	0.24	0.20	0.58	0.57
	2019	0.32	0.20	0.58	0.57
Qinghai	2015	0.38	0.35	0.82	0.74
	2016	0.39	0.34	0.81	0.73
	2017	0.41	0.40	0.83	0.78
	2018	0.43	0.40	0.84	0.76
	2019	0.39	0.36	0.87	0.82
Ningxia Hui Autonomous Region	2015	0.32	0.34	0.64	0.64
	2016	0.18	0.16	0.67	0.64
	2017	0.13	0.16	0.61	0.63
	2018	0.15	0.16	0.61	0.63
	2019	0.14	0.39	0.61	0.62
Xinjiang Uygur Autonomous Region	2015	0.17	0.21	0.36	0.36
	2016	0.18	0.17	0.39	0.37
	2017	0.17	0.10	0.37	0.29
	2018	0.19	0.36	0.37	0.32
	2019	-	0.22	-	0.44

The Gini coefficients of some districts and counties in the Xinjiang Uygur autonomous region could not be calculated because the data were not publicly available

### Equality in health resource allocation based on Theil index

The difference in the number of medical personnel among the non-impoverished counties was larger than that among the impoverished counties, with a small difference between the two county groups. From 2015 to 2019, the overall Theil Index gradually decreased, the Theil Index of impoverished counties remained stable, and the between-group Theil Index gradually decreased, except in Qinghai Province (Table 2).

**Table 2:** The Theil Index of medical personnel by population in five provinces from 2015 to 2019

Province	Year	Overall index	Within-group		Between-group
			Impoverished counties	Non-impoverished counties	
Shaanxi	2015	0.01	0.01	0.01	0.01
	2016	0.04	0.02	0.04	0.01
	2017	0.03	0.01	0.03	0.01
	2018	0.03	0.01	0.03	0.01
	2019	-0.14	0.01	-0.28	0.00
Gansu	2015	0.05	0.06	0.02	0.01
	2016	0.05	0.05	0.06	0.00
	2017	0.05	0.06	0.05	0.00
	2018	0.05	0.05	0.04	0.00
	2019	-0.27	-1.01	0.07	0.25
Qinghai	2015	0.13	0.05	0.10	0.05
	2016	0.12	0.04	0.10	0.05
	2017	0.14	0.05	0.11	0.06
	2018	0.15	0.05	0.12	0.06
	2019	0.14	0.05	0.12	0.11
Ningxia Hui Autonomous Region	2015	0.10	0.00	0.06	0.04
	2016	0.03	0.00	0.03	0.01
	2017	0.01	0.00	0.02	0.00
	2018	0.02	0.00	0.02	0.00
	2019	0.02	0.00	0.03	0.00
Xinjiang Uygur Autonomous Region	2015	0.09	0.02	0.04	0.06
	2016	0.02	0.03	0.01	0.00
	2017	0.02	0.03	0.01	0.00
	2018	0.03	0.03	0.00	0.01
	2019	-	-	-	-

The Theil Index of some districts and counties in the Xinjiang Uygur autonomous region could not be calculated because the data were not publicly available

The impoverished counties in the Gansu Province had large contribution rates, indicating a large difference in the number of medical personnel. The contribution rates of non-impoverished counties in Shaanxi Province, Qinghai Province, and the Ningxia Hui Autonomous Region were relatively large, suggesting considerable difference among non-impoverished counties in the three provinces. Shaanxi Province, Qinghai Province, and the Xinjiang Uygur Autonomous Region had large between-group contribution rates, which indicated a great difference between impoverished counties and non-impoverished counties (Table 3). From 2015 to 2019, the contribution rates of the impoverished counties gradually decreased, except for those in Gansu Province, and the between-group contribution rates gradually decreased, except for those in Qinghai Province, suggesting that the difference between them gradually narrowed.

**Table 3:** The contribution rate of medical personnel by population in five provinces from 2015 to 2019

Province	Year	Within-group		Between-group
		impoverished counties	Non-impoverished counties	
Shaanxi	2015	0.32	0.26	0.42
	2016	0.23	0.51	0.26
	2017	0.15	0.63	0.23
	2018	0.22	0.55	0.23
	2019	-0.05	1.08	-0.03
Gansu	2015	0.71	0.14	0.15
	2016	0.52	0.48	0.00
	2017	0.59	0.41	0.00
	2018	0.64	0.34	0.02
	2019	2.03	-0.11	-0.92
Qinghai	2015	0.18	0.39	0.43
	2016	0.17	0.43	0.40
	2017	0.16	0.43	0.41
	2018	0.16	0.43	0.41
	2019	0.12	0.10	0.79
Ningxia Hui Autonomous Region	2015	0.00	0.58	0.42
	2016	0.03	0.66	0.31
	2017	0.02	0.96	0.02
	2018	0.01	0.92	0.07
	2019	0.00	0.98	0.02
Xinjiang Uygur Autonomous Region	2015	0.11	0.20	0.69
	2016	0.69	0.27	0.04
	2017	0.73	0.12	0.15
	2018	0.53	0.06	0.41
	2019	-	-	-

The Theil Index of some districts and counties in the Xinjiang Uygur autonomous region could not be calculated because the data were not publicly available

The difference in the number of beds in medical institutions among non-impoverished counties was larger than that among impoverished counties; however, the difference between the two groups was not significant. From 2015 to 2019, the overall Theil Index gradually decreased, except that for those in Qinghai Province. The Theil Index of impoverished counties gradually decreased, suggesting that the difference gradually decreased. The Theil Index of non-impoverished counties remained stable, and the between-group Theil Index gradually decreased, except for those in Gansu and Qinghai provinces (Table 4).

**Table 4:** The Theil Index of beds in medical institutions by population in five provinces from 2015 to 2019

Province	Year	Overall index	Within-group		Between-group
			Impoverished counties	Non-impoverished counties	
Shaanxi	2015	0.03	0.00	0.05	0.01
	2016	0.04	0.01	0.06	0.01
	2017	0.04	0.00	0.06	0.00
	2018	0.03	0.00	0.05	0.00
	2019	-0.03	0.01	-0.06	0.00
Gansu	2015	0.00	0.03	0.04	0.04
	2016	0.03	0.04	0.02	0.00
	2017	0.04	0.03	0.04	0.00
	2018	0.03	0.02	0.04	0.00
	2019	-0.26	-0.99	0.04	0.27
Qinghai	2015	0.09	0.03	0.10	0.03
	2016	0.08	0.02	0.08	0.03
	2017	0.12	0.02	0.12	0.35
	2018	0.12	0.02	0.13	0.04
	2019	0.11	0.01	0.02	0.09
Ningxia Hui Autonomous Region	2015	0.11	0.00	0.05	0.06
	2016	0.02	0.00	0.02	0.00
	2017	0.02	0.00	0.03	0.00
	2018	0.02	0.00	0.03	0.00
	2019	0.02	0.00	0.02	0.00
Xinjiang Uygur Autonomous Region	2015	0.11	0.03	0.19	0.01
	2016	0.02	0.04	0.00	0.00
	2017	0.01	0.01	0.00	0.00
	2018	0.01	0.01	0.00	0.00
	2019	0.04	0.04	0.02	0.00

Impoverished counties in Gansu Province had large contribution rates, which indicated a large difference in the number of beds. Non-impoverished counties had large contribution rates, except for those in Gansu Province, indicating large differences among them. The between-group contribution rates were less than 40% and were the largest in Qinghai Province, suggesting that there was little difference between the two groups of counties, except for Qinghai Province. From 2015 to 2019, the contribution rates of the impoverished counties in all provinces gradually decreased, whereas those of the non-impoverished counties gradually increased. The between-group contribution rates gradually decreased, except for those in Qinghai Province (Table 5).

**Table 5:** The contribution rate of beds in medical institutions by population in five provinces from 2015 to 2019

Province	Year	Within-group		Between-group
		Impoverished counties	Non-impoverished counties	
Shaanxi	2015	0.06	0.77	0.17
	2016	0.08	0.78	0.14
	2017	0.05	0.82	0.13
	2018	0.06	0.84	0.10
	2019	-0.16	1.17	-0.01
Gansu	2015	0.45	0.42	0.13
	2016	0.64	0.30	0.07
	2017	0.51	0.48	0.01
	2018	0.41	0.57	0.01
	2019	2.08	-0.06	-1.01
Qinghai	2015	0.15	0.53	0.32
	2016	0.13	0.53	0.35
	2017	0.07	0.55	0.38
	2018	0.07	0.56	0.37
	2019	0.05	0.10	0.84
Ningxia Hui Autonomous Region	2015	0.00	0.42	0.58
	2016	0.04	0.88	0.08
	2017	0.03	0.91	0.06
	2018	0.05	0.93	0.02
	2019	0.07	0.93	0.00
Xinjiang Uygur Autonomous Region	2015	0.12	0.81	0.07
	2016	0.82	0.07	0.11
	2017	0.80	0.19	0.02
	2018	0.74	0.25	0.02
	2019	0.56	0.32	0.12

### Equality in health resource allocation based on Health Resource Agglomeration Degree

**Fig. 3:**
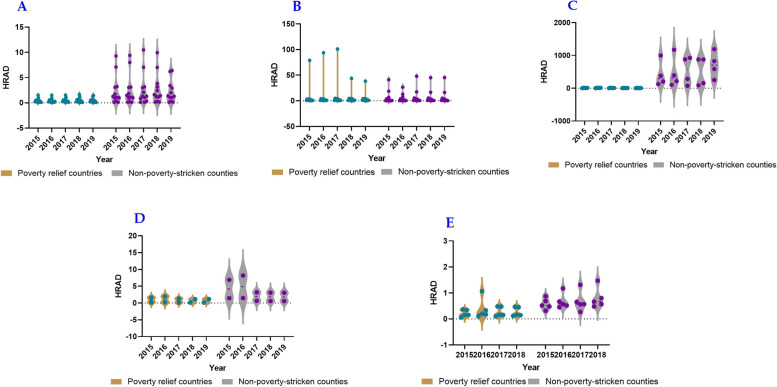
HRAD of medical and health personnel in five provinces, including Shaanxi Province (**A**), Gansu Province (**B**), Qinghai Province(**C**), Ningxia Hui Autonomous Region (**D**), Xinjiang Uygur Autonomous Region (**E**), whereas the green knots illustrated the non impoverished counties, and the purple knots illustrated the impoverished counties

**Fig. 4:**
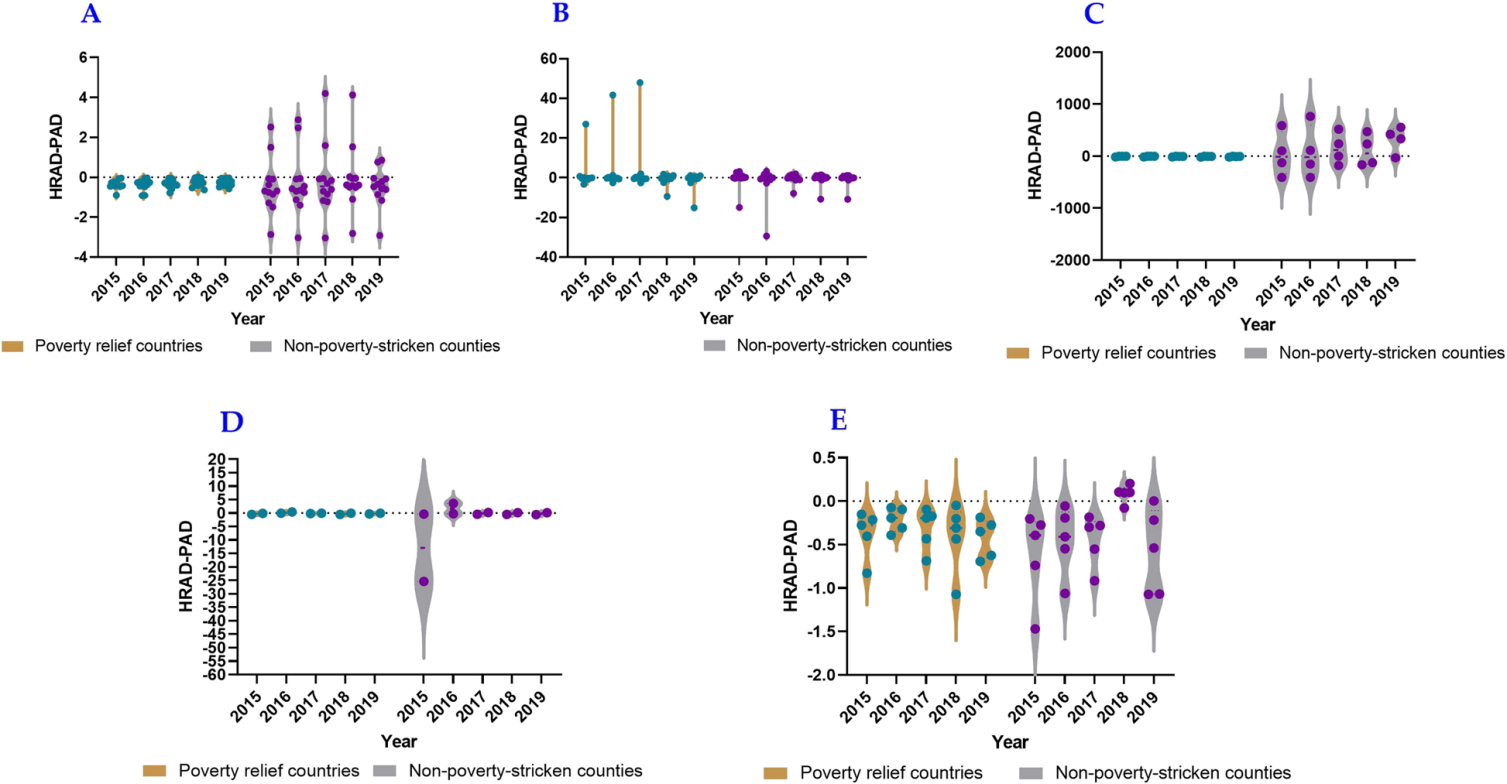
HRAD-PAD of medical and health personnel in five provinces, including Shaanxi Province (**A**), Gansu Province (**B**), Qinghai Province (**C**), Ningxia Hui Autonomous Region (**D**), Xinjiang Uygur Autonomous Region (**E**), whereas the green knots illustrated the non impoverished counties, and the purple knots illustrated the impoverished counties

The analysis of HRAD values in different provinces reveals varying patterns. In Shaanxi Province, the number of impoverished counties with HRAD values greater than 1 was considerably smaller than one of non-impoverished counties. Additionally, the HRAD-PAD values of impoverished counties were less than 0, and only 18% of non-impoverished counties had HRAD-PAD values greater than 0. This indicates an imbalanced allocation of medical personnel by area and limited accessibility of health services to residents, with impoverished counties being at a disadvantage compared to non-impoverished counties. By contrast, in Gansu Province, the number of impoverished counties with HRAD values less than one exceeded that of non-impoverished counties, and the number of impoverished counties with HRAD-PAD values less than zero was also higher. Moreover, from 2015 to 2019, both values gradually increased, suggesting a more balanced allocation of medical personnel by area and improved accessibility to health services in non-impoverished counties compared with impoverished counties. The allocation and accessibility of counties throughout the province have shown steady improvement over the years. In Qinghai Province, the HRAD values were greater than 1, and the HRAD-PAD values of the impoverished counties were lower than 0. The HRAD-PAD values of non-impoverished counties increased annually from 2015 to 2019, indicating a relatively balanced allocation of medical personnel by area, while the resources of non-impoverished counties were in surplus, resulting in a significant gap compared to impoverished counties. In the Ningxia Hui Autonomous Region, there was little difference in the HRAD values between impoverished and non-impoverished counties. However, the HRAD-PAD values of impoverished counties were all less than 0, signifying that while the allocation of medical personnel by area had minor disparities between the two groups, accessibility to health services in impoverished counties remained poor. Finally, in the Xinjiang Uygur Autonomous Region, the HRAD values were generally less than 1, and the HRAD-PAD values of impoverished counties were less than 0. Over the years, HRAD-PAD values have gradually increased, indicating an unbalanced allocation of medical personnel by area and limited accessibility to health services, with impoverished counties experiencing greater challenges than non-impoverished counties. However, there were signs of improvement in accessibility each year (Figs. 3 and 4).

In general, the allocation of medical personnel by area in non-impoverished counties exhibited better distribution than in impoverished counties, resulting in improved access to health services for residents. Counties in Qinghai Province, Northwest China, demonstrated a relatively balanced allocation of medical personnel by area. Notably, the number of medical personnel in non-impoverished counties in the Gansu and Qinghai provinces surpassed local needs, leading to a surplus of resources. However, the allocation of medical personnel in counties of the Xinjiang Uygur Autonomous Region appeared unbalanced, and both Shaanxi Province and the Xinjiang Uygur Autonomous Region faced shortages of medical personnel that were insufficient to meet the needs of their respective local populations.

**Fig. 5:**
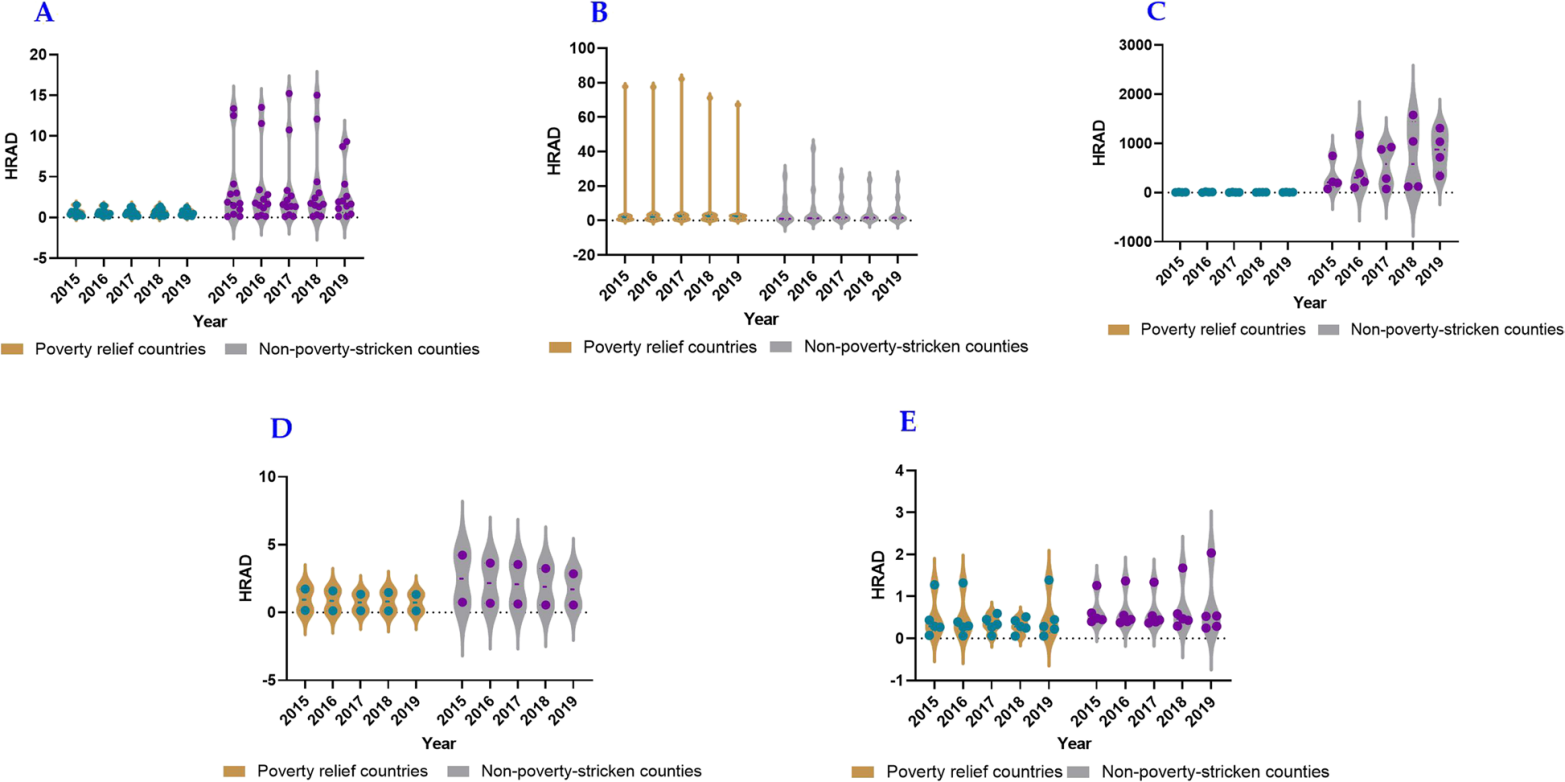
HRAD of beds in medical institutions in five provinces, including Shaanxi Province (**A**), Gansu Province (**B**), Qinghai Province (**C**), Ningxia Hui Autonomous Region (**D**), Xinjiang Uygur Autonomous Region (**E**), whereas the green knots illustrated the non impoverished counties, and the purple knots illustrated the impoverished counties

**Fig. 6:**
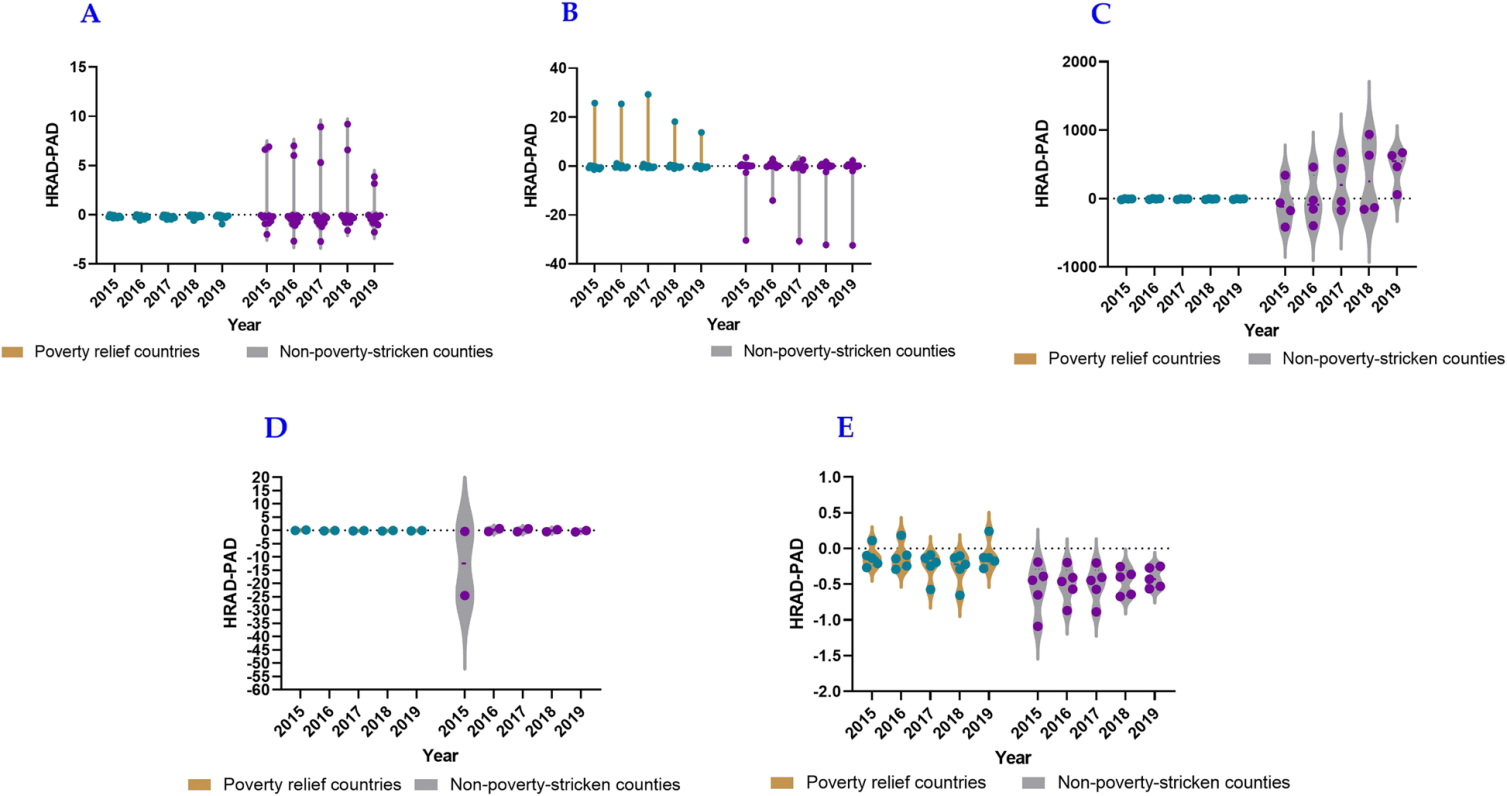
HRAD-PAD of beds in medical institutions in five provinces, including Shaanxi Province (**A**), Gansu Province (**B**), Qinghai Province (**C**), Ningxia Hui Autonomous Region (**D**), Xinjiang Uygur Autonomous Region (**E**), whereas the green knots illustrated the non impoverished counties, and the purple knots illustrated the impoverished counties

The HRAD values of most impoverished counties in Shaanxi Province were less than 1, whereas those of most non-impoverished counties were greater than 1. Additionally, the HRAD-PAD values of most counties were less than 0. This indicates an unbalanced allocation of beds in medical institutions by area in impoverished counties and poor accessibility of residents to health services. Conversely, there was a balanced allocation of beds by area in non-impoverished counties; however, residents’ access to health services remained inadequate. In Gansu Province, the number of impoverished counties with HRAD values greater than 1 was the same as that of non-impoverished counties, whereas the number of impoverished counties with HRAD-PAD values greater than 0 was higher. This suggests a better allocation of beds by area, with a smaller gap between impoverished and non-impoverished counties, while accessibility to health services in non-impoverished counties was superior to that in impoverished counties. In Qinghai Province, the HRAD values were greater than 1, and the HRAD-PAD values of impoverished counties were less than 0, indicating that the allocation by area was commendable; however, accessibility to health services in impoverished counties was poor, and non-impoverished counties had a surplus of resources. In some counties of the Ningxia Hui Autonomous Region, HRAD values were greater than 1 and HRAD-PAD values were mostly less than 0, indicating poor access to health services for their residents. For most counties in the Xinjiang Uygur Autonomous Region, the HRAD values were less than 1, and the HRAD-PAD values were mainly less than 0, indicating an unbalanced allocation of beds by area and poor accessibility to health services (Figs. 5 and 6).

In general, the allocation of beds in medical institutions by area in non-impoverished counties was better than that in impoverished counties, and accessibility to health services for residents in non-impoverished counties was better than that in impoverished counties. The beds allocated by area among counties in the Ningxia Hui Autonomous Region in Northwest China were balanced, and the number of beds in non-impoverished counties could meet the needs of local residents. There was a surplus of resources, while the beds allocated by area were unbalanced. The accessibility to health services was poor among impoverished counties in Shaanxi Province and counties in the Xinjiang Uygur Autonomous Region.

## Discussion

This comparative study focused on evaluating the equality of health resource allocation between counties that have been lifted out of impoverished and non-impoverished counties in Northwest China. In 2015, the “Decision on Winning the Tough Battle Against Poverty” was issued by the Communist Party of China (CPC) Central Committee, and the State Council, successfully achieving the poverty alleviation target as scheduled and leading to a significant victory in 2020. It is important to highlight the COVID-19 pandemic in 2020 affected the utilization of health resources. Therefore, for our analysis, we chose data from 2015 to 2019, which enabled us to assess the trends and perform a comparative analysis during the pre-pandemic period. Virtually, the.

From the data presented in this study, county health resource allocation according to population among the provinces in Northwest China demonstrated a fair distribution. However, concerning geographical areas, the allocation was found to be inequitable, and this inequality remained relatively stable throughout the fight against poverty. This finding is consistent with the results of other studies on health allocation equality in China [24, 25], especially in Northwest China, which covers a vast territory with a sparse population. The possible reasons for the little change in equality were as follows: First, the equality of county health resource allocation had been high, with difficulty increasing. Second, compared to healthcare resources, the Health and Medical Assistance Program for Poverty Alleviation focused more on reducing the burden of medical expenses on the poor, which has been proven to be the fastest and most direct way to alleviate poverty caused by illness.

However, despite the high equality in county level health resource allocation among provinces in Northwest China, a gap exists between Western and Eastern China [2]. This study focuses only on the quantity of health resources, not quality, and the equality of quantity does not indicate the equality of quality. Truthfully, the quality of health services and efficiency of health service utilization has been gradually improving after the Health and Medical Assistance Program for Poverty Alleviation, although the utilization efficiency of health resources in Western China was lower than that in Eastern China [26–28]. Consequently, when allocating resources, the government should adopt a comprehensive approach that considers various factors, including but not limited to population and geographical area. Simultaneously, it is crucial to consider the quality and efficiency of healthcare services. Merely focusing on the equality of quantity cannot fully indicate the overall equality of healthcare resource allocation.

It is worth noting that the county health resource allocation of Qinghai Province is the least equitable within Northwest China, while allocation according to geographical area is the most equitable in the Xinjiang Uygur Autonomous Region. Qinghai Province has 44 county(district)-level regions, but only four districts are non-impoverished in Xining City. Thus, we randomly selected four counties that were lifted out of poverty to compare their health resource allocations. Additionally, Xining City’s GDP(1286.41 billion yuan) accounts for nearly half the entire GDP of Qinghai Province(2865.23 billion yuan), indicating an imbalance in economic development across various cities (prefectures). Previous studies have shown that economically developed areas have more concentrated health resources [29], which may explain why many health resources are concentrated in Xining City. This also accounts for the issue of surplus healthcare resources in Qinghai.Meanwhile, health resource allocation according to geographical area is the most equitable in the Xinjiang Uygur Autonomous Region, similar to the results of related studies [30].

The Theil Index contribution rate can help identify the causes of inequality in health resource allocation. Compared with impoverished counties, the equality of non-impoverished counties’ health resource allocations is worse. Since 2015, China has made significant investments in combating poverty, resulting in a more rapid increase in health resource investments in impoverished counties than in non-impoverished counties. Certain non-impoverished counties have received less attention in terms of health-related investments, which encompasses the formulation of policies, allocation of funds, implementation efficiency, and impact assessment across various stages [31, 32]. Significantly, when analyzing development trends, it becomes evident that the gap between the two categories of counties has narrowed.

Especially in the western region, there may be considerable distance between high-quality health resources and patient homes, leading to challenges in accessing such resources. This underscores the importance of considering distance and population density when allocating health resources. A comparison reveals that geographical access to health resources in impoverished counties is generally lower than that in non-impoverished counties. The disparity between the two categories of counties is particularly pronounced in Shaanxi and Qinghai Provinces, among the five provinces in Northwest China. Moreover, in some instances, these counties have surplus health resources. Poverty due to illness has two important causes: labor shortages caused by diseases and high medical costs [33]. The direct impact of diseases, particularly chronic and severe illnesses, on family labor participation and productivity is substantial. This is largely due to the prevalence of informal sector labor participation in developing countries and rural areas, especially in impoverished counties. In such contexts, the health status of family members can directly influence the economic stability of the household, as illness or disability may result in the loss of primary income sources. Access to health resources is pivotal in preventing the disease processing. This may explain why geographical access to health resources in impoverished counties is generally low. Such counties in Shaanxi Province are distributed mainly in the southern region, most of which have inconvenient transportation and poor economies caused by the mountainous terrain. Qinghai Province is similar to Shaanxi Province in this regard, suggesting that health resources should be allocated according to local conditions.

The HRAD-PAD value could help to better understand whether health resource allocation could meet residents’ needs. Our results showed that county-level health resource allocation could not fully meet this demand. On this issue, little difference can be observed between the two counties. The number of health technicians per 1000 people has reached a significant level(7.74). The level of medical services in China is also ranked eighth globally, and the UHC effective coverage index is 70, indicating that the country has much progress to achieve. However, in terms of quality and structure, health resource allocation requires improvement [34]. This situation further underscores the necessity of the government's initiative, “Healthy China 2030,” which emphasizes the improvement of population health while continuously enhancing the level of health resources [35].

## Conclusion

Our findings reveal significant differences in equality among the five provinces in Northwest China, which were mainly derived from non-impoverished counties. Although equality is gradually improving, the number of health resources in poverty-relief counties remains lower than that in non-impoverished counties. The findings indicate that, in addition to addressing the equality of healthcare resources, it is imperative to consider the quality and utilization of healthcare services.

### Limitations

Owing to data limitations, some results from the Qinghai Province present significant differences. In Qinghai Province, only the four administrative districts of Xining City are not classified as poverty-stricken counties, which restricts the sampling size. Additionally, we did not utilize data from the recent three years because of the potential impact of the COVID-19 pandemic on health resource allocation. In future research, additional data sources are required to further evaluate the equality of health resources, including nationwide data, and gain a more comprehensive understanding.

Equality in health resource allocation alone cannot demonstrate the total utilization of health resources. Furthermore, the quality of healthcare resources is also not measured in this study.Thus, future research should evaluate both the equality of health resource allocation and the efficiency of the utilization of health resources.The quantity as well as the quality of healthcare resources should also be taken into account. It is insufficient to rely solely on the number of beds as an indicator of resource availability, as the mere presence of beds does not automatically ensure the quality of related infrastructure, human resources, and medical services. Other parameters such as bed occupancy rate, turnover rate, nurse-to-bed ratio, and equipment investment per bed should be considered to form a comprehensive understanding of healthcare resource capacity.

The Gini coefficient does not capture the total amount or per capita level of resources, but rather focuses solely on distribution disparities without accounting for their origins. In contrast, the Theil index addresses variations within and between regions or groups, serving as a valuable complement to the Gini coefficient. However, it is more susceptible to data sensitivity, and missing data may result in biased outcomes.

Overall, this study acknowledges its limitations and highlights potential avenues for future research to overcome these constraints and improve our understanding of equality in health resource allocation.

### Supplementary Information

Supplementary Material 1.

## Data Availability

The datasets generated during the current study are available in the *Statistical Report of National Economic and Social Development* of each county, *China Statistical Yearbook(County-level)*and the *Chinese Statistical Yearbook*. The *Statistical Report of National Economic and Social Development *can be found on the government website of each county, and the yearbook can be found on the National Bureau of Statistics website. These datasets are available from the corresponding authors on request.
