# Supplementary material for: Equality of healthcare resource allocation between impoverished counties and non-impoverished counties in Northwest China: a longitudinal study
Source: BMC Health Serv Res. 2024 Jul 22;24:830. doi: 10.1186/s12913-024-11312-5 (PMC11265348; doi:10.1186/s12913-024-11312-5)
Supplement: Supplementary file 1 — Supplementary Material 1. [file 12913_2024_11312_MOESM1_ESM.docx]

Supplementary material-The sample list of impoverished and non-impoverished counties

| Number | Province(Autonomous Region) | Types | County(District) |
| --- | --- | --- | --- |
| 1 | Shaanxi Province | impoverished counties | Yintai District |
| 2 | Shaanxi Province |  | Chengcheng County |
| 3 | Shaanxi Province |  | Yaozhou District |
| 4 | Shaanxi Province |  | Yanchang County |
| 5 | Shaanxi Province |  | Mian County |
| 6 | Shaanxi Province |  | Shanyang County |
| 7 | Shaanxi Province |  | Langao County |
| 8 | Shaanxi Province |  | Yang County |
| 9 | Shaanxi Province |  | Foping County |
| 10 | Shaanxi Province |  | Danfeng County |
| 11 | Shaanxi Province |  | Ningshan County |
| 12 | Shaanxi Province |  | Chenggu County |
| 13 | Shaanxi Province | non-impoverished counties | Wangyi District |
| 14 | Shaanxi Province |  | Sanyuan County |
| 15 | Shaanxi Province |  | Binzhou District |
| 16 | Shaanxi Province |  | Luochuan County |
| 17 | Shaanxi Province |  | Wugong County |
| 18 | Shaanxi Province |  | Qishan County |
| 19 | Shaanxi Province |  | Tongguan County |
| 20 | Shaanxi Province |  | Liquan County |
| 21 | Shaanxi Province |  | Ganquan County |
| 22 | Shaanxi Province |  | Mei County |
| 23 | Shaanxi Province |  | Feng County |
| 24 | Shaanxi Province |  | Hantai District |
| 1 | Gansu Province | impoverished counties | Gulang County |
| 2 | Gansu Province |  | Jingyuan County |
| 3 | Gansu Province |  | Hezheng County |
| 4 | Gansu Province |  | Linxia City |
| 5 | Gansu Province |  | Lintao County |
| 6 | Gansu Province |  | Longxi County |
| 7 | Gansu Province |  | Hezuo City |
| 8 | Gansu Province |  | Huan County |
| 9 | Gansu Province |  | Jingchuan County |
| 10 | Gansu Province |  | Zhangjiachuan Hui Autonomous County |
| 11 | Gansu Province |  | Kang County |
| 12 | Gansu Province | non-impoverished counties | Jinchuan District |
| 13 | Gansu Province |  | Zhenyuan County |
| 14 | Gansu Province |  | Minle County |
| 15 | Gansu Province |  | Anning District |
| 16 | Gansu Province |  | Xigu District |
| 17 | Gansu Province |  | Honggu District |
| 18 | Gansu Province |  | Linze County |
| 19 | Gansu Province |  | Yongchang County |
| 20 | Gansu Province |  | Pingchuan District |
| 21 | Gansu Province |  | Minqin County |
| 22 | Gansu Province |  | Huangyuan County |
| 1 | Qinghai Province | impoverished counties | Guide County |
| 2 | Qinghai Province |  | Huangzhong County |
| 3 | Qinghai Province |  | Datong Hui-Tu Autonomous County |
| 4 | Qinghai Province |  | Chengdong District |
| 5 | Qinghai Province | non-impoverished counties | Chengzhong District |
| 6 | Qinghai Province |  | Chengxi District |
| 7 | Qinghai Province |  | Chengbei District |
| 8 | Qinghai Province |  | Longde County |
| 1 | Ningxia Hui Autonomous Region | impoverished counties | Yanchi County |
| 2 | Ningxia Hui Autonomous Region |  | Litong District |
| 3 | Ningxia Hui Autonomous Region | non-impoverished counties | Qingtongxia City |
| 4 | Ningxia Hui Autonomous Region |  | Balikun County |
| 1 | Xinjiang Uygur Autonomous Region | impoverished counties | Hetian County |
| 2 | Xinjiang Uygur Autonomous Region |  | Cele County |
| 3 | Xinjiang Uygur Autonomous Region |  | Atushi City |
| 4 | Xinjiang Uygur Autonomous Region |  | Aktao County |
| 5 | Xinjiang Uygur Autonomous Region |  | Wenquan County |
| 6 | Xinjiang Uygur Autonomous Region | non-impoverished counties | Wensu County |
| 7 | Xinjiang Uygur Autonomous Region |  | Shaya County |
| 8 | Xinjiang Uygur Autonomous Region |  | Xinhe County |
| 9 | Xinjiang Uygur Autonomous Region |  | Baicheng County |
| 10 | Xinjiang Uygur Autonomous Region |  | Baicheng County |
